# Identification of HIV-1 Tat-Associated Proteins Contributing to HIV-1 Transcription and Latency

**DOI:** 10.3390/v9040067

**Published:** 2017-04-01

**Authors:** Maxime Junior Jean, Derek Power, Weili Kong, Huachao Huang, Netty Santoso, Jian Zhu

**Affiliations:** 1 Department of Microbiology and Immunology, University of Rochester Medical Center, Rochester, NY 14642, USA; Maxime_Jean@urmc.rochester.edu (M.J.J.); derekpow@buffalo.edu (D.P.); Weili_Kong@urmc.rochester.edu (W.K.); Huachao_Huang@urmc.rochester.edu (H.H.); 2 Department of Biochemistry and Biophysics, University of Rochester Medical Center, Rochester, NY 14642, USA

**Keywords:** HIV, Tat, transcription, latency, proteomics, PLATO, protein interaction, ribosome display

## Abstract

Human immunodeficiency virus type 1 (HIV-1) Tat is a virus-encoded trans-activator that plays a central role in viral transcription. We used our recently developed parallel analysis of in vitro translated open reading frames (ORFs) (PLATO) approach to identify host proteins that associate with HIV-1 Tat. From this proteomic assay, we identify 89 Tat-associated proteins (TAPs). We combine our results with other datasets of Tat or long terminal repeat (LTR)-associated proteins. For some of these proteins (NAT10, TINP1, XRCC5, SIN3A), we confirm their strong association with Tat. These TAPs also suppress Tat-mediated HIV-1 transcription. Removing suppression of HIV-1 transcription benefits the reversal of post-integrated, latent HIV-1 proviruses. We demonstrate that these transcriptionally suppressing TAPs contribute to HIV-1 latency in Jurkat latency (J-LAT) cells. Therefore, our proteomic analysis highlights the previously unappreciated TAPs that play a role in maintaining HIV-1 latency and can be further studied as potential pharmacological targets for the “shock and kill” HIV-1 cure strategy.

## 1. Introduction

Tat is a virus-encoded regulatory protein that is essential for human immunodeficiency virus type 1 (HIV-1) replication. Although Tat possesses multiple functions, it is primarily involved in the transcription of integrated HIV-1 proviruses. Once the host cellular transcription factors nuclear factor kappa B (NF-κB) and Nuclear factor of activated T-cells 1 (NFAT1) bind to the enhancer region of the HIV-1 5’ long terminal repeat (LTR) promoter, they recruit histone acetyltransferase (HAT) to acetylate histones and stimulate viral gene expression. Tat protein is therefore produced, which induces a positive feedback loop to amplify HIV-1 transcription. Tat associates with the RNA polymerase II (Pol II) pre-initiation complex (PIC), and plays a role in both initiation and elongation [1]. Tat increases PIC formation on HIV-1 5’ LTRs and stabilizes the complex during elongation [1,2]. Trans-activation responsive (TAR) element—a pre-terminated 59-nucleotide stem-bulge-loop RNA hairpin structure—further binds to Tat at the HIV-1 5’ LTR promoter, which displaces 7SK small nuclear ribonucleoprotein (snRNP) and activates the positive elongation factor b (P-TEFb) positioned next to the paused Pol II. Activated P-TEFb phosphorylates the C-terminal domain (CTD) of Pol II as well as the negative elongation factors DRB sensitivity inducing factor (DSIF) and negative elongation factor (NELF) to promote transcriptional elongation. Further studies show that additional cellular factors are involved for Tat-P-TEFb to fully activate HIV-1 transcription. Affinity purifications for Tat and P-TEFb lead to the identification of ELL1/2, AFF1/4, ENL, and AF9 as novel components that associate with the Tat-P-TEFb complex and form the super elongation complex (SEC) [3,4]. Paradoxically, Tat associates with the host proteins that suppress HIV-1 transcription [5], indicating Tat’s complicated functions of either activating or silencing gene expression in a context-dependent manner. Tat also regulates the transcription of host genes, for instance, interferon-stimulated genes and inflammation-related cytokines [6]. Besides transcription, Tat can be secreted from infected cells and bind with surface receptors of other cells, such as integrins, CXCR4, CD26, HSPG and LRP [7]. This leads to Tat endocytosis, and cytosolic Tat binds with host proteins in cytoplasm and triggers various cell responses [8].

Tat is a small protein (86–101 amino acids), containing the N-terminal, proline-rich, cysteine-rich core, basic, and the C-terminal glutamine-rich regions. The structural studies indicate that Tat alone lacks a defined three-dimensional folding with a stable conformation and is intrinsically a disordered protein [9]. However, Tat is still able to form tight binding with partners, such as TAR element and cyclin T1, since its structural plasticity allows Tat to adopt different but specific conformations upon interaction with different partners and therefore to assemble the high-affinity complexes [10]. Tat undergoes numerous post-translational modifications, such as phosphorylation, acetylation, methylation, and ubiquitination [11], which further increases the multiplicity of the Tat interaction with host proteins by providing Tat with more flexible confirmation changes and/or contacting interfaces. Overall, these features make Tat a versatile viral protein suitable for establishing multiple interactions with many host proteins to execute diversified tasks, which benefits HIV-1 propagation and/or causes HIV-1 pathogenesis. Several proteomic studies have been conducted to identify Tat- or LTR-associated proteins, mostly based on the affinity purification (AP) coupled with mass spectrometry (MS) approach [12,13,14]. However, the AP/MS approach has intrinsic problems, such as heterogeneous protein expressions, noisy cellular backgrounds, and identification of both direct and indirect interactions. To mitigate these issues, we recently developed a proteomic method, PLATO (parallel analysis of translated open reading frames (ORFs)), which combines the in vitro ribosome display of full-length proteins with the analysis of protein enrichment through high-throughput DNA sequencing [15,16]. PLATO provides an alternative, complementary platform to study protein interactions in vitro with high reliability and sensitivity. We hope to compile a complete list of Tat- or LTR-associated proteins, from which we will be able to identify novel TAPs that may regulate HIV-1 transcription and latency.

## 2. Methods and Materials

### 2.1. Cells and Plasmids

HEK293 and HEK293T cells were maintained in Dulbecco’s Modified Eagle Medium (DMEM) supplemented with 10% Fetal Bovine Serum (FBS). J-LAT A2 was obtained from the National Institutes of Health (NIH) AIDS reagent repository, and cultured in Roswell Park Memorial Institute (RPMI) 1640 medium with 10% FBS. pQCXIP-FLAG-Tat and HIV-LTR-luciferase vectors were described previously [17]. pcDNA-V5-TAP was constructed through Gateway^®^ *att*L-containing entry clone and an *att*R-containing destination vector (LR) cloning using pDONR223-TAP entry vector and pcDNA-DEST40 destination vector (Life Technologies, Carlsbad, USA).

### 2.2. Small Molecules

Prostratin, suberoylanilide hydroxamic acid (SAHA), and JQ1 were purchased (Santa Cruz Biotechnology, Dallas, TX, USA). Compounds were used at the following concentrations: prostratin (1 μM), SAHA (0.5 μM), JQ1 (1 μM). Drug-treated cells were cultured in the presence of compounds for 24 h and subjected to flow cytometry assays on a FACS Calibur flow cytometer (Becton Dickinson, Franklin Lakes, NJ, USA), and results acquired using BD CellQuest software and analyzed using FlowJo vx.0.7 program. Puromycin for selecting stably-transduced cells was purchased (Fisher Scientific, Hampton, VA, USA). 

### 2.3. Viruses

Lentviruses were produced by transfecting pAPM-TAP-short hairpin RNA (shRNA) plasmids in HEK293T cells using TransIT®-293 Transfection Reagent (Mirus, Madison, WI, USA). Cell supernatants containing lentiviruses were harvested and filtered through 0.45 μm filter (Millipore, Billerica, MA, USA). Viruses were stored in aliquots at −80 °C for later use. To generate cell lines for stable expression of shRNAs, lentiviruses were transduced in cells (HEK293, J-LAT A2). At 72 h of post transduction, puromycin (1 μg/mL) was added to the medium for stable selection. 

### 2.4. Antibodies

The following antibodies were used in this study: rabbit anti-KA5/Tip60 (Thermo Scientific, Waltham, MA, USA); rabbit anti-SIN3A (Bethyl, Montgomery, AL, USA); mouse anti-JMJD1A (Santa Cruz Biotechnology, Santa Cruz, CA, USA); mouse anti-V5 (Invitrogen, Carlsbad, CA, USA); rabbit anti-FLAG (Rockland, Limerick, USA); mouse anti-FLAG (Sigma, St. Louis, MO, USA); unlabeled rabbit and mouse IgGs (Southern Biotech, Birmingham, AL, USA).

### 2.5. shRNAs

shRNAs targeting TAPs were cloned into the pAPM lentiviral vector following the reported protocol [17]. The targeted sequences of TAP-shRNAs: shNAT10, 5′-GAA AGA CCC TCA GTG ACG ACC T-3′; shTINP1, 5′-GCC AGC GTC ATG CTG AGA AAA T-3′; shXRCC5, 5′-GAC TGG GAG TTC TAA CAA AAC A-3′; shSIN3A, 5′-GAA AGA TGG TGT ATG TGA TCA A-3′; shTH1L, 5′-GAA AGC GAG TGA GCA TCA ATA A-3′; shNME1, 5′-GCC GGC TTG TGG TTT CAC CCT G-3′; shIFI6, 5′-GGG CAG CGT CGT CAT AGG TAA T-3′; shNT, 5′-CAC AAA CGC TCT CAT CGA CAA G-3′.

### 2.6. PLATO

PLATO for this study followed previously described protocol [15,16], except that recombinant glutathione S-transferase (GST)-Tat or GST immobilized on glutathione beads were used as the bait molecules. Ranking of ORF enrichment from PLATO assay is average of two repeats.

### 2.7. Luciferase Reporter Assays

To measure the effect of TAPs on HIV-1 LTR promoter activity, HEK293 cells stably expressing pAPM-TAP-shRNA were co-transfected with pcDNA-Tat, HIV-LTR-luciferase, and pRL-TK-renilla vectors using turbofect reagent (Thermo Scientific, Waltham, MA, USA). At 48 h post-transfection, luciferase unit (LU) was measured using Dual Glo^®^ Luciferase Assay System (Promega, Madison, WI, USA) and normalized to Renilla signal. All results were collected on a Luminoskan Ascent Microplate Luminometer (Thermo Scientific, Waltham, MA, USA). HEK293 cells were transiently transfected with pcDNA-V5-TAP for the gain-of-function luciferase assays. 

### 2.8. Co-Immunoprecipitation (co-IP)

Co-IP assays followed the previously described protocol [18] with minor changes. In brief, HEK293 cells in 10 cm tissue culture dishes were transfected with pQCXIP-FLAG-Tat and pcDNA-V5-TAP. At 48 h post-transfection, cells were harvested and lysed in 1 mL 1× RIPA buffer (50 mM Tris-HCl (pH 7.4), 150 mM NaCl, 1% NP-40, 0.25% Na deoxycholate, 1 mM EDTA, and protease inhibitor cocktail (Pierce)). Cell lysate was sonicated briefly and debris was spun down. The supernatant was transferred to a new tube and pre-cleared by incubating with 50 μL protein A/G beads (Pierce) for 2 h at 4 °C. The beads were removed, and the lysate was split equally for incubating with 2 ug of mouse anti-V5 or IgG (mIgG) control antibody with rotation for overnight at 4 °C. Then 25 μL of protein A/G beads were added to each sample and incubated for another 2 h at 4 °C. The beads were washed three times with 1× RIPA buffer and precipitated. Protein samples were eluted in 1× NuPAGE^®^ LDS Sample Buffer (Life Technologies, Carlsbad, CA, USA), and analyzed by sodium dodecyl sulfate polyacrylamide gel electrophoresis (SDS-PAGE) and western blots for FLAG-Tat. To investigate the interaction of FLAG-Tat and endogenous TAP, pQCXIP-FLAG-Tat was transfected in HEK293 cells. Cell lysate was immunoprecipitated using mouse/rabbit endogenous anti-TAP or mouse/rabbit IgG antibody. Protein samples were analyzed by SDS-PAGE and western blots for FLAG-Tat. 

### 2.9. Real-Time Quantitative Polymerase Chain Reaction (qPCR)

To measure shRNA mediated gene depletion, total RNA was extracted from cells using the RNeasy Mini Kit (Qiagen, Mainz, Germany) and subsequently reverse transcribed using random hexamers (0.1 μM) and iScript™ cDNA Synthesis Kit (Bio-Rad, Hercules, CA, USA). The gene-specific primers (0.1 μM each) were mixed with reverse transcribed cDNA templates and iTaq™ Universal SYBR^®^ Green Supermix (Bio-Rad, Hercules, USA). The qPCR reaction was performed on the CFX Connect™ Real-Time PCR Detection System (Bio-Rad), in a 20 μL volume using the following program: 95 °C for 1 m, 40 cycles of 95 °C for 15 s and 60 °C for 30 s. Glyceraldehyde-3-phosphate dehydrogenase (GAPDH) was used as an internal control. The primer sequences for TAP qPCRs: NAT10 forward, 5′-GCT CAG TCG GCT CTT CTC TT-3′, NAT10 reverse, 5′-TTC ACA ACT TTG CGG ATG AT-3′; TINP1 forward, 5′-GGA AGT CCC TCT GCC TAA AG-3′; TINP1 reverse, 5′-CCA TCT CCA ACA AAG CAC AC-3′; XRCC5 forward, 5′-GTT GCA CTT TCC TCC CTG AT-3′, XRCC5 reverse, 5′-GCC GAC TTG AGG ATT AGC TC-3′; SIN3A forward, 5′-CAT TGC TGT TCC AAT TGT CC-3′, SIN3A reverse, 5′-CTG GTG GTC CAG AGA CTT CA-3′; TH1L forward, 5′-TTG TGT TGC AAC GAG AAC AA-3′, TH1L reverse, 5′-TGC AGC TGA AAG TAC CTT GG-3′; NME1 forward, 5′-GAG ACC AAC CCT GCA GAC TC-3′, NME1 reverse, 5′-CCT TCT CTG CAC TCT CCA CA-3′; IFI6 forward, 5′-GGT CTG CGA TCC TGA ATG GG-3′, IFI6 reverse, 5′-TCA CTA TCG AGA TAC TTG TGG GT-3′ [19]; GAPDH forward, 5′-GCC TCT TGT CTC TTA GAT TTG GTC-3′; GAPDH reverse,5′-TAG CAC TCA CCA TGT AGT TGA GGT-3′. 

## 3. Results

### 3.1. PLATO Analysis of Tat-Associated Proteins

**Figure 1:**
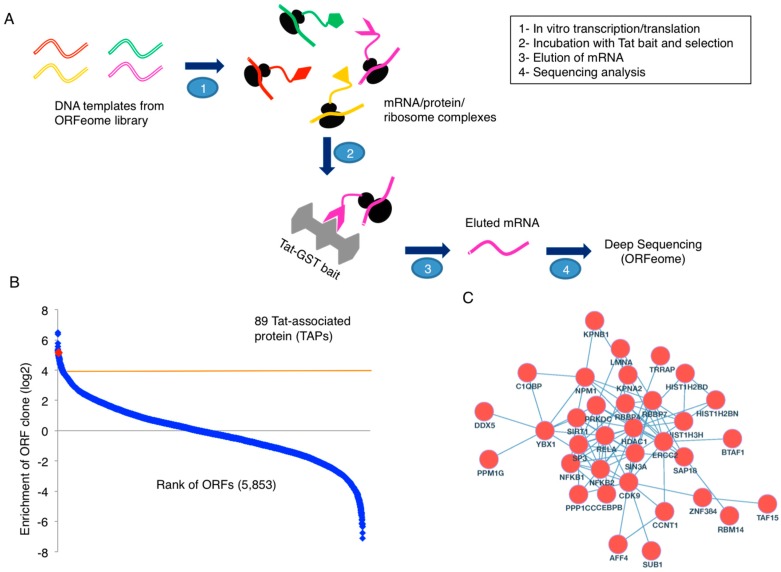
Proteomic analysis of Tat associated proteins. (**A**) A schematic view for parallel analysis of translated open reading frames (ORFs) (PLATO) assays using purified glutathione S-transferase (GST)-Tat or GST proteins immobilized on glutathione beads. (**B**) PLATO identification of novel Tat-associated proteins. After data filtering, around 5853 open reading frames (ORFs) were ranked according to the ratio of log_2_[GST-Tat/GST] (from the largest to the smallest). Eighty-nine Tat-associated proteins (TAPs) were identified using the cutoff log_2_[GST-Tat/GST] ≥4. The position of a new TAP, TINP1, was highlighted. (**C**) We further combined our list of TAPs from PLATO analysis with two published datasets of TAPs, one dataset of TAPs from the National Center for Biotechnology Information (NCBI) human immunodeficiency virus type 1 (HIV-1) Human Interaction Database, and one dataset of long terminal repeat (LTR)-associated proteins. There are in total 566 unique Tat- or LTR-associated proteins with 34 identified from at least two proteomic screens. The protein-protein interaction (PPI) network for the 34 proteins in the Search Tool for the Retrieval of Interacting Genes (STRING) database was visualized.

To dissect the molecular functions of HIV-1 Tat, we applied our recently developed PLATO proteomic approach to identify host proteins that associate with Tat (Figure 1A). Specifically, we carried out the in vitro transcription/translation of host cellular proteins from a human ORFeome library. This allowed for the formation of mRNA/protein/ribosome complexes that were then incubated with glutathione beads carrying immobilized GST-Tat or GST alone as a control (Figure S1). Following mRNA elution and deep sequencing analysis, we identified 89 Tat-associated proteins (TAPs) (cutoff: log_2_[GST-Tat/GST] ≥4) (Figure 1B). 

We further combined our list of TAPs with two published datasets of TAPs [12,13], one dataset of TAPs from the National Center for Biotechnology Information (NCBI) HIV-1 Human Interaction Database, and one dataset of LTR-associated proteins [14], mostly generated from the MS approach. There are in total 566 unique Tat- or LTR-associated proteins (Table S1). Among them, 34 proteins are identified from at least two proteomic screens (Table S1). There are 9078 and 87 protein-protein interactions (PPIs) for 566 and 34 proteins, respectively, listed in the Search Tool for the Retrieval of Interacting Genes (STRING) database (with the setting of protein interactions from experiments, literature, and a confidence score 0.7 or higher) [20] (Table S2). The PPI networks are visualized using Cytoscape [21] (Figure 1C and Figure S2). Gene ontology (GO) analysis was performed for 566 and 34 proteins using BiNGO [22]. Statistically significant GO terms (*p* < 0.001) are mostly related to transcription factor activity (Table S1), reconfirming the role of Tat as a major transcriptional regulator, although it has several other functions. In fact, this group includes the components of recently identified super elongation complex (SEC), such as P-TEFb (CDK9/CCNT1) and AFF4, which is required for proper HIV-1 transcription elongation [23,24]. Furthermore, this subset of proteins also contains known transcriptional factors required for HIV-1 gene expression, such as subunits of NF-κB (NFKB1, NFKB2, RELA), Transcription Factor II (TFII) holoenzyme (BTAF1, ERCC2, TAF15), as well as several others (CEBPB, SP3, SUB1, TAF15, TRRAP, YBX1). Interestingly, multiple subunits of the SIN3/HDAC transcriptional repressor complex (HDAC1, SAP18, SIN3A, SIRT1) are in this subset, suggesting that Tat indeed can recruit transcriptional suppressors to aid in the establishment of HIV-1 latency. These Tat interactions warrant further studies, including the assembling mechanisms and interaction kinetics. As a matter of fact, both the GO terms for transcription activator and transcription repressor activity are statistically significant (*p* < 0.0001) for 566 and 34 proteins (Table S1), indicating that Tat would be required for assembling protein interactions to either activate or silence HIV-1 viral transcription.

### 3.2. Validation of Tat-TAP Interactions

**Figure 2:**
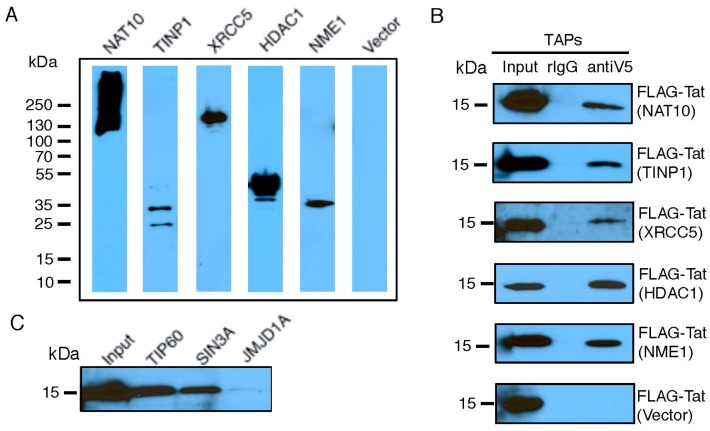
Validation of Tat-TAP interactions. (**A**,**B**) Interaction of co-transfected V5-TAPs and FLAG-Tat. A set of V5-tagged TAPs or empty vector was co-transfected with FLAG-Tat in HEK293 cells. Expression of V5-TAPs was confirmed by immunoblottings using an anti-V5 antibody (**A**). Cell lysates were prepared for immunoprecipitations (IPs) using an anti-V5 antibody. Co-IPed FLAG-Tat was determined by immunoblottings using an anti-FLAG antibody (**B**). (**C**) Interaction of endogenous TAPs and transfected FLAG-Tat. FLAG-Tat was transfected in HEK293 cells. Cell lysates were prepared for IPs using antibodies against endogenous TAPs. Co-IPed FLAG-Tat was determined by immunoblottings using an anti-FLAG antibody.

We selected a set of TAPs for validation of their interactions with Tat, based on their molecular function as transcriptional modulators (Table S3) and their ranking in our earlier RNAi screens [25]. FLAG-Tat and V5-TAP were co-expressed in HEK293 cells. The presence of V5-TAP in cell lysate was verified (Figure 2A and Figure S3). Protein co-immunoprecipitations were performed using a V5 antibody. We confirmed a strong interaction with Tat for five TAPs (NAT10, TINP1, XRCC5, HDAC1, and USP11), a weak Tat interaction for two TAPs (IFI6, ZNF384), and no Tat interaction for one TAP (RANGAP1) (Figure 2B and Figure S3). We included NME1 or an empty vector as a positive or negative control for the Tat interaction, respectively. Among the tested TAPs, TINP1 and IFI6 were never reported as Tat-associated proteins from earlier studies. Consequently, they are newly found TAPs. Furthermore, NAT10, XRCC5, and USP11 were suggested as TAPs in earlier proteomic studies (13,14,15); however, they were never confirmed to interact with Tat in cells. Consequently, our immunoprecipitation studies provide the first in vivo evidence for these TAPs (NAT10, XRCC5 and USP11) as bona fide Tat-associated proteins. Lastly, for certain TAPs, such as SIN3A, we were unable to clone them for overexpression. Therefore, we determined the interaction of FLAG-Tat with an endogenous level of these TAPs. Protein co-immunoprecipitations were performed using the TAP-specific antibody. Using this approach, we confirmed the strong Tat interaction for one previously described TAP (SIN3A) (Figure 2C). We included TIP60 or JMJD1A as a positive or negative control, respectively. NAT10 is a membrane-associated histone acetyltransferase [26]. It interacts with BRD4, an inhibitory protein of HIV-1 transcription, which would explain NAT10’s suppressive activity [27]. TINP1 (NSA2) is a nucleolus protein that regulates cell proliferation and cell cycle through the inhibition of p53 and p21 expression [28,29], indicating that TINP1 may possess transcription-suppressing activities. XRCC5 is the 80-kilodalton subunit of the Ku heterodimer protein (Ku80) that functions in the repair of DNA double-strand breaks. Ku proteins also play a role in transcriptional regulation [30,31]. SIN3A is a key component of the SIN3/HDAC transcriptional repressor complex [32], which may explain the impact of SIN3A knockdown on HIV transcription. 

### 3.3. TAP-Mediated Suppression of HIV-1 LTR Promoter

The key function of Tat is to regulate HIV-1 transcription. We next examined whether TAPs play a role in HIV-1 transcription. Specifically, we selected TAPs that would elicit restrictive function in HIV transcription based on their known activity (Table S3). shRNA-targeting TAPs (NAT10, TINP1, XRCC5, SIN3A, IFI6) were cloned in a pAPM lentiviral vector and transduced into HEK293 cells. shTH1L and NME-1 were used as positive and negative controls, respectively. Data is represented relative to the non-targeting shRNA (shNT) control.

**Figure 3:**
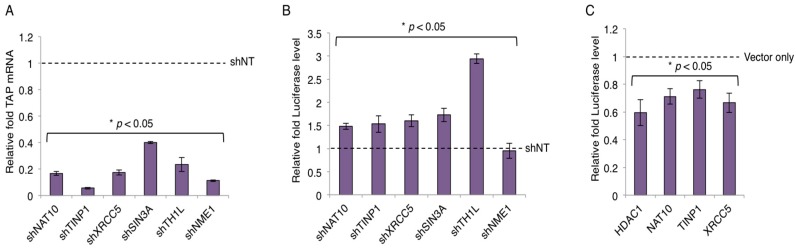
TAP-mediated suppression of HIV-1 LTR promoter. (**A**) Knockdown of TAP-specific short hairpin RNAs (shRNAs). pAPM-TAP-shRNAs were transduced in HEK293 cells for stable selection. TAP depletion was determined by reverse transcription coupled with qPCR using the TAP-specific primers. (**B**) Impact of TAP depletion on HIV LTR promoter activity. pAPM-TAP-shRNA stably transduced HEK293 cells were transfected with pcDNA-Tat, HIV-LTR-luciferase, and pRL-TK-renilla vectors. Luciferase signal was measured and normalized to renilla signal. Depletion of TH1L (NELFCD) or NME1 by shRNA served as a positive or negative control, respectively. Results are presented as fold induction compared to non-targeting shRNA (shNT) control (mean ± s.e.m. *p* < 0.05, Student’s *t*-test). (**C**) Effects of overexpression of V5-TAPs in HEK293 cells on HIV-1 LTR. HEK293 cells were transiently transfected with V5-tagged TAPs (HDAC1, NAT10, TINP1, XRCC5) and subjected to the HIV LTR-luciferase assays. Luciferase signal was measured and analyzed as described in panel B.

The gene knockdown of TAPs was confirmed by qPCR and the knock-down efficiency was 60% or greater across selected TAPs (Figure 3A and Figure S3). Next, HEK293 cells stably expressing TAP-shRNAs were subjected to the HIV LTR-luciferase assays. Depletion of TAPs (NAT10, TINP1, XRCC5, SIN3A) modestly increased the luciferase expression (~1.5 fold or more) with the presence of HIV-1 Tat protein expressed from a pcDNA vector (Figure 3B). However, the depletion of IFI6 showed no obvious effect on the luciferase expression (Figure S4). We also overexpressed the cDNAs of TAPs that were successfully cloned (Figure 2A) and determined their impact on LTR promoter activity. Specifically, HEK293 cells transiently transfected with V5-tagged TAPs (HDAC1, NAT10, TINP1, XRCC5) were subjected to the HIV LTR-luciferase assays. Consistently, overexpression of these TAPs modestly suppressed the luciferase expression (~25%–45% reduction) with the presence of HIV-1 Tat protein expressed from a pcDNA vector (Figure 3C). The small inhibitory effect may be due to the already existing expression of endogenous TAPs. 

### 3.4. Contribution of TAP to HIV-1 Latency

The restrictive effects of NAT10, TINP1, XRCC5, and SIN3A on HIV-1 transcription led us to speculate their contributing role in the maintenance of HIV-1 latency. Consequently, we determined whether the depletion of TAPs benefits the reversal of latent HIV-1. We used the J-LAT A2 cells that harbor the latently infected HIV “LTR-Tat-IRES-green fluorescent protein (GFP)” minigenome. J-LAT A2 cells stably expressing TAP-shRNAs were treated with dimethyl sulfoxide (DMSO), JQ1 (a BETi), or SAHA (a HDACi), and subjected to flow cytometry analysis for the quantification of GFP-positive cells. The depletion of TH1L (NELFCD) or NME1 by shRNA served as the positive or negative control, respectively. Relative fold of GFP-positive cells as compared to shNT control was used to represent the data.

**Figure 4:**
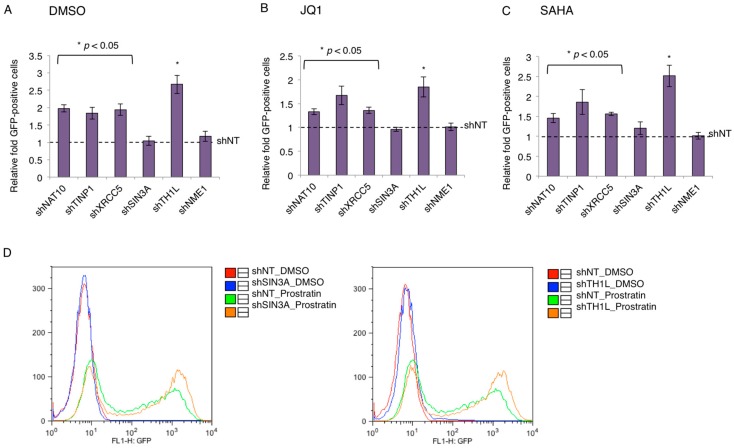
Contribution of TAP to HIV-1 latency. (**A**–**C**) Depletion of TAPs reverses HIV-1 latency in J-LAT A2 cells. pAPM-TAP-shRNAs were transduced in J-LAT A2 cells for stable selection. Cells were treated with DMSO (**A**), JQ1 (**B**), or SAHA (**C**). HIV reactivation in above cells was measured by flow cytometry by gating for the green fluorescent protein (GFP)-positive cells. Depletion of TH1L (NELFCD) or NME1 by shRNA served as the positive or negative control, respectively. The GFP-positive percentage of TAP-depleted J-LAT A2 cells was normalized to those transduced with shNT control (mean ± s.e.m. *p* < 0.05, Student’s *t*-test). (**D**) Depletion of SIN3A and TH1L (NELFCD) synergizes with prostratin to reverse HIV-1 latency in J-LAT A2 cells. pAPM-SIN3A/TH1L/NT-shRNAs were transduced in J-LAT A2 cells for stable selection. Cells were treated with dimethyl sulfoxide (DMSO) or prostratin. HIV-1 reactivation in above cells was measured by flow cytometry by gating for the GFP-positive cells. One of three individual experiments is represented.

At the basal level (DMSO), the percentage of the GFP-positive cell population for shNT-transduced J-LAT A2 cells was 2.06%, and all TAPs except for SIN3A showed a two-fold induction in GFP-positive cells as compared to control shNT (Figure 4A). In the induced conditions using the different latency-reversing agents (LRAs), JQ1 or SAHA, the percentage of the GFP-positive cell population for shNT-transduced J-LAT A2 cells was 25.9% and 6.48%, respectively. The depletion of TAPs (NAT10, TINP1, XRCC5) increased the percentage of GFP-positive cells by ~1.5- to 2.0-fold relative to the control shNT in these conditions, except for SIN3A (Figure 4B,C). Although the depletion of SIN3A yielded no beneficial effect in JQ1- or SAHA-treated J-LAT A2 cells, we found that SINA3A depletion did synergize with a different LRA, prostratin (PKC agonist), to reactivate HIV-1 in J-LAT A2 cells, generating a similar effect as shTH1L (Figure 4D). 

## 4. Discussion

It is generally believed that HIV-1 trans-activator Tat recruits host proteins required for promoting viral transcription [24]. However, recent studies show that Tat also associates with host proteins that suppress viral transcription and facilitate HIV-1 latency [5]. These studies are not contradictory, but rather reflect the comprehensive roles of Tat in assembling different machineries to either activate or suppress viral transcription. The association of Tat with host transcriptional complexes is also dynamic and subjected to regulation depending on the stage of HIV-1 infection. Thorough investigation of Tat protein interactions is important to enhance the understanding of Tat’s cellular functions. However, the identified TAPs from different studies overlap with each other poorly. This is not unusual for proteomic studies, considering a lot of variants across these studies. Nonetheless, we can still extrapolate useful information from these proteomic data in order to achieve an overall view of the different HIV-1 Tat host protein partners. 

In an effort to identify new TAPs and compile a comprehensive list of TAPs, we first used our recently described PLATO assay (Figure 1A) and combined our results with similar proteomic studies. We identified 89 TAPs through our study, and a total of 566 unique Tat-interacting proteins or LTR-associated proteins were deduced once all proteomics datasets were combined (Table S1). Interestingly, for the total 556 proteins, most of statistically significant GO terms (*p* < 0.001) are relevant to transcription factor activity (Table S1), indicating that the main function of Tat is still in gene transcription, although Tat may play other roles. From the list of 556 proteins, a subset of 34 TAPs were identified by at least two proteomic screens pointing to the importance of these host factors for Tat activity (Table S1).

From the combined list of Tat- or LTR-associated proteins, we selected a few proteins for validation (i.e., NAT10, TINP1, XRCC5, SIN3A). From our studies, we successfully identified a set of TAPs that strongly associate with Tat (Figure 2) and suppress HIV-1 LTR promoter activity (Figure 3). We validated NAT10 which was originally identified as a TAP from a Tat interactome study [12] and TINP1 which was exclusively identified from our PLATO studies. Another TAP that we further validated was XRCC5. Earlier studies of XRCC5 showed that it associates with the HIV LTR promoter [14,33] and HIV-1 TAR RNA [34]. However, we are the first group to report that XRCC5 also interacts directly with Tat (Figure 2B). Additionally, our finding of XRCC5 as a suppressive factor of HIV-1 transcription is consistent with others [33]. The TAP-SIN3A was also considered for validation studies. Interestingly, previous studies of SIN3A showed that it associates with both HIV-1 Tat [12] and LTR [14]. It is plausible that transcription-suppressing TAPs associate with Tat and negatively regulate HIV-1 LTR promoter activity. Moreover, they might be recruited at a specific time to facilitate the establishment of HIV-1 latency. 

Indeed, the depletion of transcription-suppressing TAPs enhances the reversal of latently infected HIV-1 proviruses in the J-LAT–A2 cell model, albeit at a moderate effect (Figure 4). It generally only a yielded mild beneficial effect (about two-fold). Nonetheless, this observation is similar to effects seen by others in genetic studies of single HIV-1 host transcription repressors [35,36,37]. For instance, Jiang et al. have shown that the depletion of the transcription factor c-myc in the J89GFP HIV-1 latency cell line led to an approximate of 2.5-fold induction of GFP+ cells [36]. Consequently, these mild effects seen when a single factor is knocked down seem to suggest that the silencing of HIV-1 transcription for latency establishment requires the cooperation of multiple host factors and machineries. Furthermore, a two-fold increase is considered to be clinically significant for the “shock and kill” strategy to cure HIV [38]. Interestingly, most TAPs (NAT10, TINP1, XRCC5) showed beneficial effects when their depletion was coupled with LRAs (JQ1, SAHA), except for SIN3A as compared to the shNT control (Figure 4). However, the depletion of SIN3A by RNAi generates the HIV latency-reversing effect only when cells are induced by the PKC agonist prostratin (Figure 4). This result can provide insights into how the SIN3/HDAC complex contributes to HIV-1 latency. 

Overall, our proteomic studies of TAPs have identified new and previously described host factors that associate with Tat. Moreover, we have shown that some of these TAPs can impose an inhibitory effect on HIV-1 transcription and may contribute to the maintenance of HIV-1 latency. Our initial results in the simple but well-defined J-LAT–A2 cell model of HIV-1 latency support this idea. Nevertheless, further examination of these TAPs’ effects on HIV-1 latency is warranted and will be the subject of future studies. On the other hand, this present study also establishes the PLATO method as a great tool to study host-virus protein interactions. Here, we used HIV Tat as a platform to study such interactions; however, it is conceivable that this technique can be used for viral proteins from other viruses. The fact that we identified both new and previously described TAPs argues that our assay is highly reliable. In addition, we have demonstrated that our hits also had a functional role in HIV-1 infection. Consequently, in the ever-growing concern for emerging pathogens such as Zika or Ebola, our technique will be a great additional tool to elucidate important virus-host interactions to help tailor proper drug development.

## Supplementary Materials

The following are available online at www.mdpi.com/1999-4915/9/4/67/s1, Figure S1: **Recombinant GST-Tat or GST protein**. GST-Tat and GST recombinant proteins were expressed in *E. coli* bacteria and purified. Cell lysates and purified proteins were ran on SDS-PAGE gel and visualized by coomassie staining, Figure S2: **PPI network for 566 Tat- or LTR-associated proteins**. TAPs identified from PLATO analysis were combined with two published datasets of TAPs, one dataset of TAPs from the NCBI HIV-1 Human Interaction Database, and one dataset of LTR-associated proteins. There are in total 566 unique Tat- or LTR-associated proteins. The protein-protein interaction network shown here is a visualization of these 556 proteins listed in the STRING database, Figure S3: **Interactions of IFI6 and ZNF384 with HIV-1 Tat**. (A) Expression of the following V5 tagged proteins: IFI6, ZNF384, USP11 and RANGAP1. (B) Interactions of co-transfected V5-IFI6 and V5-ZNF384 with FLAG-Tat. Cell lysates were prepared for immunoprecipitations (IPs) using an anti-V5 antibody. Co-IPed FLAG-Tat was determined by immunoblotting using an anti-FLAG antibody, Figure S4: **Depletion of IFI6 has no effect on HIV LTR promoter activity**. (A) shRNA targeting IFI6 was cloned in pAPM lentiviral vector and transduced into HEK293 cells. The gene knockdown was confirmed by qPCR. Data normalized to non-targeting shRNA (shNT). (B) HEK293 cells stably expressing shIFI6 were subjected to the HIV LTR-luciferase assay. However, depletion of IFI6 showed no obvious effect on the luciferase expression. Results are normalized to shNT, Table S1: Combination of Tat- or LTR-associated proteins from multiple proteomic studies, Table S2: PPIs for 556 and 34 Tat- or LTR-associated proteins in STRING database, Table S3: Description of TAPs that were selected for validation of Tat interaction.
