# Supplementary material for: Identification of HIV-1 Tat-Associated Proteins Contributing to HIV-1 Transcription and Latency
Source: Viruses. 2017 Apr 1;9(4):67. doi: 10.3390/v9040067 (PMC5408673; doi:10.3390/v9040067)
Supplement: Supplementary file 1 [file viruses-09-00067-s001.tgz › Supplementary revised/SupplementaryMaterials/TableS3.docx]

| *Genes* | *Description* |
| --- | --- |
| NAT10 | NAT10 is a membrane-associated histone acetyltransferase [[1](#_ENREF_1)]. NAT10 interacts with BRD4, an inhibitory protein of HIV-1 transcription, which may implicate this host factor as having suppressive effects on HIV-1 transcription [[2](#_ENREF_2)]). |
| TINP1 | TINP1 (or NSA2) is a nucleolus protein. Previous studies have implicated this factor as a regulator of cell proliferation and cell cycle through inhibition of p53 and p21 expression [[3](#_ENREF_3), [4](#_ENREF_4)]. |
| XRCC5 | XRCC5 is the 80-kilodalton subunit of the Ku heterodimer protein (Ku80) that functions in repair of DNA double-strand break. Ku proteins also play a role in transcriptional regulation [[5](#_ENREF_5), [6](#_ENREF_6)]. |
| HDAC1 | HDAC1 is a member of chromatin remodeling family known as histone deacetylase. Their primary function involves gene transcription repression. HDAC1 is recruited by different host factors (e.g. NF-kb; c-myc) to repress HIV-1 transcription and maintain HIV-1 latency in reservoir cells [[7](#_ENREF_7), [8](#_ENREF_8)]. |
| USP11 | USP11 belongs to the family of deubiquitinase enzymes. USP11 prevents TNFa-mediated IkBa degradation and plays a key role in the down-regulation of NF-Kb activation [[9](#_ENREF_9)]. |
| IFI6 | IFI6 is a type I interferon stimulated gene [[10](#_ENREF_10)]. IFI6 has the anti-apoptotic effects in dengue-infected cells through the inhibition of pro-apoptotic factor, Bax [[11](#_ENREF_11)]. Additionally, IFI6 also promotes HCV RNA replication *in vitro* [[12](#_ENREF_12)]. |
| SIN3A | SIN3A is one of the two isoforms of the SIN3 gene. It is a complex of several proteins that act as a major transcriptional regulator of gene expression. SIN3A is known to associate with HDAC1 and other repressors to silence gene expression via histone deacetylation [[13](#_ENREF_13)]. |
| ZNF384 | ZNF384 is a zinc finger protein that function as a host transcription factor. It is notably associated with development of acute leukemia through translocation with other genes such as TAF-15 [[14](#_ENREF_14)]. |
| RANGAP1 | RANGAP1 is the activating protein for Ran, a Ras GTPase, involved in nuclear trafficking [[15](#_ENREF_15)]. Ran is involved in nuclear accumulation of Integrase during HIV-1 infection [[16](#_ENREF_16)]. |

**Table S3**

* TAPs labeled in red are subjected to functional studies.

**References**

1. Chi, Y.H., et al., *Histone acetyltransferase hALP and nuclear membrane protein hsSUN1 function in de-condensation of mitotic chromosomes.* J Biol Chem, 2007. **282**(37): p. 27447-58.

2. Alsarraj, J., et al., *BRD4 short isoform interacts with RRP1B, SIPA1 and components of the LINC complex at the inner face of the nuclear membrane.* PLoS One, 2013. **8**(11): p. e80746.

3. Zhang, H., et al., *NSA2, a novel nucleolus protein regulates cell proliferation and cell cycle.* Biochem Biophys Res Commun, 2010. **391**(1): p. 651-8.

4. Li, W., et al., *A novel human TINP1 gene promotes cell proliferation through inhibition of p53 and p21 expression.* Oncol Rep, 2013. **30**(4): p. 1848-52.

5. Willis, D.M., et al., *Regulation of osteocalcin gene expression by a novel Ku antigen transcription factor complex.* J Biol Chem, 2002. **277**(40): p. 37280-91.

6. Li, G.C., et al., *Suppression of heat-induced hsp70 expression by the 70-kDa subunit of the human Ku autoantigen.* Proc Natl Acad Sci U S A, 1995. **92**(10): p. 4512-6.

7. Williams, S.A., et al., *NF-kappaB p50 promotes HIV latency through HDAC recruitment and repression of transcriptional initiation.* EMBO J, 2006. **25**(1): p. 139-49.

8. Jiang, G., et al., *c-Myc and Sp1 contribute to proviral latency by recruiting histone deacetylase 1 to the human immunodeficiency virus type 1 promoter.* J Virol, 2007. **81**(20): p. 10914-23.

9. Sun, W., et al., *USP11 negatively regulates TNFalpha-induced NF-kappaB activation by targeting on IkappaBalpha.* Cell Signal, 2010. **22**(3): p. 386-94.

10. Itzhaki, J.E., et al., *Targeted breakage of a human chromosome mediated by cloned human telomeric DNA.* Nat Genet, 1992. **2**(4): p. 283-7.

11. Qi, Y., et al., *IFI6 Inhibits Apoptosis via Mitochondrial-Dependent Pathway in Dengue Virus 2 Infected Vascular Endothelial Cells.* PLoS One, 2015. **10**(8): p. e0132743.

12. Chen, S., S. Li, and L. Chen, *Interferon-inducible Protein 6-16 (IFI-6-16, ISG16) promotes Hepatitis C virus replication in vitro.* J Med Virol, 2016. **88**(1): p. 109-14.

13. Grzenda, A., et al., *Sin3: master scaffold and transcriptional corepressor.* Biochim Biophys Acta, 2009. **1789**(6-8): p. 443-50.

14. Martini, A., et al., *Recurrent rearrangement of the Ewing's sarcoma gene, EWSR1, or its homologue, TAF15, with the transcription factor CIZ/NMP4 in acute leukemia.* Cancer Res, 2002. **62**(19): p. 5408-12.

15. Matunis, M.J., J. Wu, and G. Blobel, *SUMO-1 modification and its role in targeting the Ran GTPase-activating protein, RanGAP1, to the nuclear pore complex.* J Cell Biol, 1998. **140**(3): p. 499-509.

16. Fassati, A., *HIV infection of non-dividing cells: a divisive problem.* Retrovirology, 2006. **3**: p. 74.
