# Supplementary figures and images for: Identification of HIV-1 Tat-Associated Proteins Contributing to HIV-1 Transcription and Latency
Source: Viruses. 2017 Apr 1;9(4):67. doi: 10.3390/v9040067 (PMC5408673; doi:10.3390/v9040067)

# Figure S1

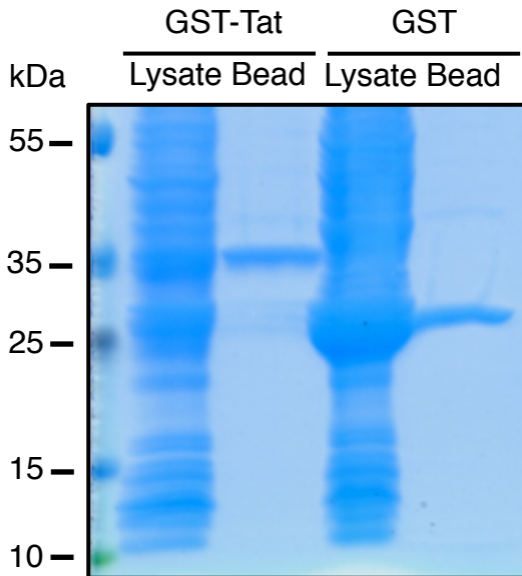

## Figure S2

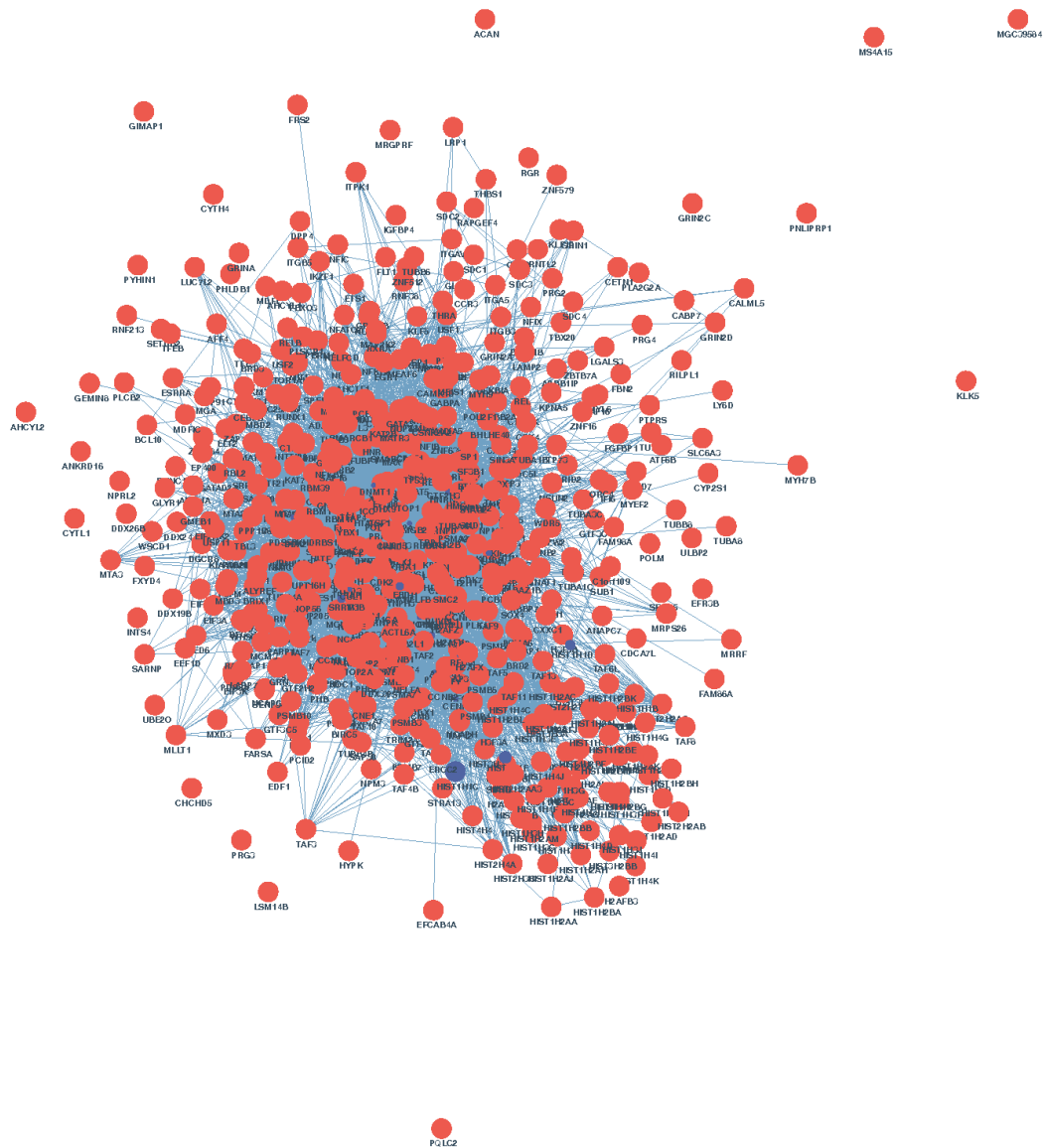

**Figure S3**

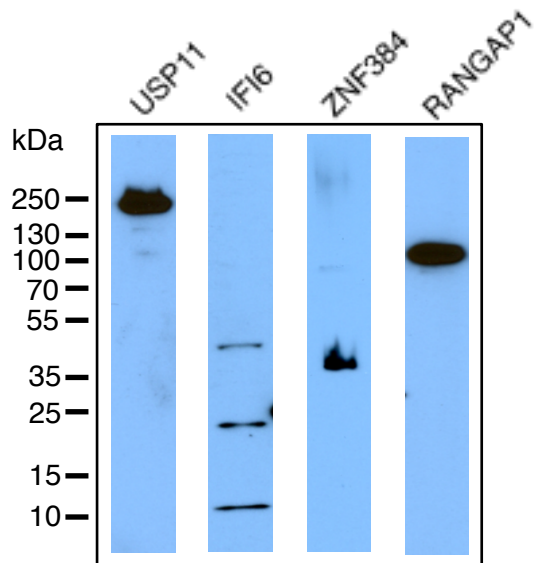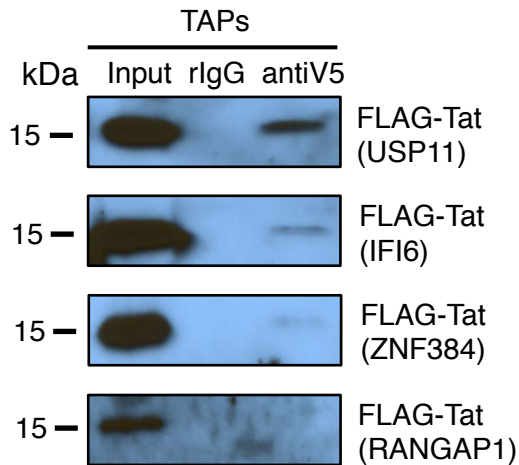

**Figure S4**

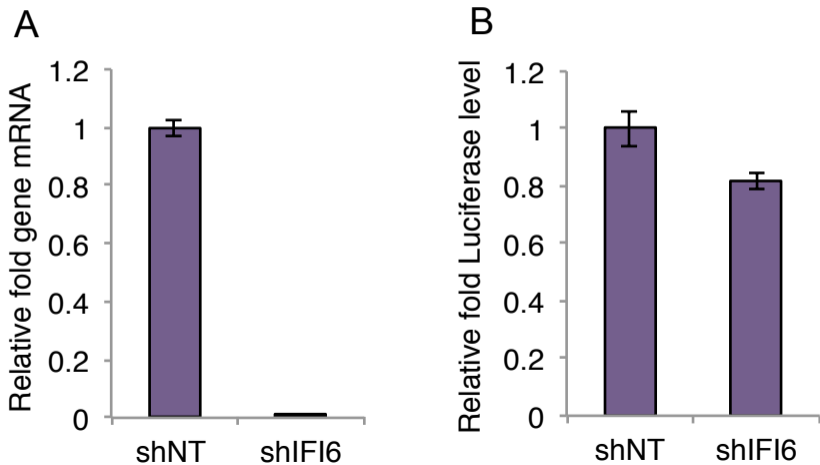

Supplement: Supplementary file 1 [file viruses-09-00067-s001.tgz › Supplementary revised/SupplementaryMaterials/SupplementaryFigures.pdf]
